# The Pyrethroid Pesticide Esfenvalerate Suppresses the Afternoon Rise of Luteinizing Hormone and Delays Puberty in Female Rats

**DOI:** 10.1289/ehp.11119

**Published:** 2008-05-13

**Authors:** Michelle D. Pine, Jill K. Hiney, Boyeon Lee, W. Les Dees

**Affiliations:** Department of Veterinary Integrative Biosciences, Texas A&M University, College Station, Texas, USA

**Keywords:** esfenvalerate, hypothalamus, luteinizing hormone, puberty, pyrethroid pesticides

## Abstract

**Background:**

One of the most widely used classes of insecticides is the synthetic pyrethroids. Although pyrethroids are less acutely toxic to humans than to insects, *in vitro* studies have suggested that pyrethroids may be estrogenic.

**Objectives:**

We assessed pubertal effects by orally administering 0.5, 1.0, and 5.0 mg/kg/day of the type II pyrethroid esfenvalerate (ESF) to female rats beginning on postnatal day (PND) 22 until vaginal opening. ESF administration suppresses serum estradiol and delays pubertal onset.

**Materials and methods:**

To assess possible hypothalamic and/or pituitary effects, animals received 0.5 or 1.0 mg/kg ESF or corn oil on PNDs 22–29. On PND30, we drew three blood samples (200 μL) from each rat at 15-min intervals beginning at 1000 hours, and again at 1500 hours. To test hypothalamic responsiveness, after the third afternoon sample, all animals received an intravenous injection of *N*-methyl-d,l-aspartic acid (NMA; 40 mg/kg), and then we drew two more samples. We performed a second experiment as above except that animals received luteinizing hormone–releasing hormone (LHRH; 25 ng/rat) to test pituitary responsiveness.

**Results:**

Basal levels of luteinizing hormone (LH) in the afternoon hours were higher in control animals than in animals treated with 1.0 mg/kg ESF (*p* < 0.05). Furthermore, NMA- and LHRH-stimulated LH release was similar in control and ESF-treated animals, indicating that both hypothalamic and pituitary responsiveness, respectively, were unaffected.

**Conclusions:**

Although the hypothalamus is able to respond to exogenous stimuli, absence of a normal afternoon rise in LH would indicate a hypothalamic deficit in ESF-treated animals.

Pesticides are used worldwide to control both agricultural and household pests. In 2001, the United States alone used approximately 122 million pounds of insecticides, and 12% of those compounds were for home and garden use [U.S. Environmental Protection Agency (EPA) 2001]. One of the most frequently used classes of pesticides is the synthetic pyrethroids (Roberts and Hutson 1999). They represented approximately one-fourth of the worldwide market for insecticides in 1998 (Casida and Quistad 1998), and their use has continued to grow.

Pyrethroid pesticides are the synthetic analogs of the naturally occurring toxin pyrethrin, which is derived from the flowers of *Chrysanthemum cinerariaefolium*. Pyrethroids exert their toxic action by binding to the voltage-dependent sodium channel in nervous tissue and prolonging the open phase (Soderlund et al. 2002; Vijverberg and van den Bercken 1990). Although these pesticides have been modified to be more photostable, more lipophilic, and more toxic than pyrethrin, they are considerably less toxic to mammals than are other classes of insecticides, such as organochlorines, organophosphates, and carbamates. Because of their low acute human toxicity, pyrethroids are widely used to control insects in and around homes (Freeman et al. 2004) and day care facilities. Tulve et al. (2006) detected two permethrin isomers and 13 different pyrethroids, including esfenvalerate (ESF), in floor wipe samples taken from 168 day care centers in the United States.

In addition to household exposures, children may also consume pyrethroids in their diet. Permethrin and fenvalerate residues have been detected in selected baby food [Food and Drug Administration (FDA) 2005). Additionally, permethrin, cyfluthrin, cypermethrin, and deltamethrin have been detected in human breast milk in women living in an area of South Africa in which pyrethroids were used for malaria control. (Bouwman et al. 2006). Pyrethroid metabolites have also been measured in urine samples taken from children with no known previous exposure (Heudorf and Angerer 2001). However, that study found no correlation between levels of permethrin (the only pyrethroid found in analyzed household dust samples) and levels of pyrethroid metabolites in the urine, leading those researchers to conclude that the exposure route was primarily dietary. However, in a study measuring pyrethroid metabolites in the urine of children 3–11 years of age, Lu et al. (2006) found that self-reported residential pyrethroid use was significantly associated with metabolite levels, whereas changing the diet from conventional to organic was not. Thus, pyrethroid exposure in this group of children is likely to have occurred primarily through the environment.

Only a few published reports of dietary intake levels of the different pyrethroids are available. The average daily intake for permethrin for a man weighing 70 kg was estimated at 3.2 μg/day, which is less than the acceptable daily intake of 50 μg/kg/day [Centers for Disease Control and Prevention (CDC) 2005], but no average daily intakes for children were listed. However, in a recent study Bouwman et al. (2006) calculated daily intakes of permethrin (13.6 μg/kg), cyfluthrin (73.4 μg/kg), and deltamethrin (13.3 μg/kg) for infants based on levels measured in breast milk of women living in three separate regions of Africa. Although calculated intakes of permethrin and cyfluthrin were below the currently established acceptable daily intake levels, those of deltamethrin exceeded the acceptable daily intake by 3.3 μg/kg.

Although the overall incidence of fatalities and severe poisonings due to pyrethroids is lower than that of the organophosphates, several *in vitro* studies have indicated that pyrethroids may have estrogenic activity, causing them to be placed on the U.S. EPA’s list of possible endocrine disruptors (Colborn et al. 1993; U.S. EPA 1997). Fenvalerate has been shown to induce proliferation and increase the expression of the estradiol (E_2_)-inducible gene *pS2* and the proto-oncogene *Wnt10B* in MCF-7 breast cancer cells (Chen et al. 2002; Go et al. 1999; Kasat et al. 2002). Lemaire et al. (2006) showed that fenvalerate was able to increase transcription via estrogen receptor-α (ER-α ) in stably transfected HELN cells. Other studies have shown conflicting data in which fenvalerate had no effect on pS2 mRNA expression, ER binding, or ER expression (Kim et al. 2004). Furthermore, fenvalerate has been shown to inhibit MCF-7 BUS (a variant MCF-7 cell line) proliferation in the presence of 17β-E_2_, leading to speculation that it is a possible antiestrogen (Kim et al. 2004).

Most of the data suggesting the estrogenic action of the type II pyrethroids have come primarily from *in vitro* studies using human cancer cell lines. When synthetic pyrethroids, including fenvalerate, were tested by other screening assays, such as the luciferase reporter gene assay (Andersen et al. 2002), yeast two-hybrid assay, and competitive ligand-binding assay using fluoromone ES1, the results were negative for estrogenic activity (Saito et al. 2000). Additionally, *in vivo* screening tests [Organisation for Economic Co-operation and Development (OECD) 1997] that assessed the estrogenic and androgenic effects of the pyrethroids fenvalerate, ESF, and permethrin found no significant changes in the accessory sex glands or uterine weights of castrated adult male and female rats, respectively, after treatment with the pesticides (Kunimatsu et al. 2002), thus calling into question the conclusions of estrogenic activity drawn from the *in vitro* data.

However, none of the *in vivo* studies has evaluated the neuroendocrine effects of oral exposure to low doses of type II pyrethroids in immature animals. Because children and adolescents are exposed to pyrethroids and because of the conflicting data regarding the possible endocrine-disrupting capability of these pesticides, we wanted to assess the effects of short-term oral administration of a low dose of a type II pyrethroid on the onset of female puberty and on the levels of pubertal hormones *in vivo*.

We chose to evaluate the pyrethroid pesticide ESF [benzeneacetic acid, 4-chloro-α-(1-methylethyl)-, cyano(3-phenoxyphenyl)methyl ester] because it is used both in the home (Ortho Bug-B-Gon; Scotts Miracle-Gro Company, Marysville, OH) and on a wide variety of crops, including fruits, vegetables, and nuts [Extension Toxicology Network (EXTOXNET) 1996].

## Material and Methods

### Animals

For these experiments, we used immature female Sprague-Dawley rats raised in our colony at the Texas A&M University Department of Comparative Medicine. The animals were housed under controlled conditions of photoperiod (lights on at 0600 hours; lights off at 1800 hours) and temperature (23°C), with *ad libitum* access to food and water. All procedures used were approved by the University Animal Care and Use Committee and in accordance with the National Institutes of Health *Guide for the Care and Use of Laboratory Animals* (Institute of Laboratory Animal Resources 1996). Animals were treated humanely and with regard for alleviation of suffering. Rats were bred and allowed to deliver their pups normally. On postnatal day (PND) 2, pups were culled from individual litters, if necessary, so that the litter size for each dam was 10–12 pups total, with at least 5–6 females per litter. We used female pups from 50 separate litters for the experiments. For all experiments, we randomly assigned littermates to treatment groups.

### Pesticide

We purchased ESF (Asana 98%, lot 306-118A, expiration date July 2009; and lot 328-113B, expiration date September 2010) from Chem Service, Inc. (West Chester, PA) and dissolved it in corn oil (Sigma Chemical Co., St. Louis, MO) for the dosing studies. We mixed dosing solution every other day and stored it at room temperature protected from light. Control animals received an equal volume of corn oil.

### Effect of ESF exposure on puberty-related hormones and the onset of puberty

For the first experiment, we administered 0.5, 1.0, or 5.0 mg/kg/day ESF by gastric gavage to female pups beginning on PND22 and continuing until vaginal opening (VO) occurred. We used a random-block experimental design. We randomly assigned pups from multiple litters to either a control or treatment group, so that each ESF treatment group had its own control group. Dosing occurred in the morning, and dosing volume was 10.0 mL/kg (0.1 mL/ 10.0 g) body weight. Once VO occurred, we performed vaginal lavage daily and observed cytology until first diestrus (D1), which indicates sexual maturity (Nyberg et al. 1993). In the second experiment, we randomly assigned PND22 female pups from multiple litters to 0, 0.5, 1.0, or 5.0 mg/kg ESF, gavaged them as previously described until PND29. All animals were killed by decapitation between 0900 and 1100 hours in a random order and were confirmed to be prepubertal by well-established criteria (Dees and Skelley 1990). Briefly, all animals had small uteri, no intraluminal fluid, and closed vaginas. Trunk blood was collected, centrifuged it at 4°C, and stored at −80°C until assayed for luteinizing hormone (LH), follicle-stimulating hormone (FSH), and E_2_.

### Effect of ESF on morning and afternoon basal levels of LH and hypothalamic responsiveness to N-methyl-d,l-aspartic acid (NMA)

Animals were dosed with 0, 0.5, or 1.0 mg/kg ESF from PND22 to PND29. The 29-day-old female rats were then anesthetized with 2.5% tribromoethanol (Aldrich, Milwaukee, WI) and Silastic cannulas were inserted into the right external jugular vein of each rat (Harms and Ojeda 1974). The next day, an extension of tubing was attached to each cannula and flushed with heparinized saline (100 IU/mL). After a 1-hr acclimation period, three basal blood samples (200 μL) were drawn at 15-min intervals from each freely moving animal, beginning at 1000 hours. All animals received an equal volume of heparinized saline to replace blood volume. The animals were left undisturbed until 1500 hours, at which time three more samples were taken at 15-min intervals. After the third afternoon sample, we conducted a hypothalamic response test by administering a single intravenous injection of NMA (Sigma) at a dose of 40 mg/kg (Gore et al. 1996; Nyberg et al. 1993). NMA is known to induce release of luteinizing hormone–releasing hormone (LHRH) in rats (Bourguignon et al. 1990; Nyberg et al. 1993; Smyth and Wilkinson 1994; Urbanski and Ojeda 1987) and primates (Claypool et al. 2000; Dissen et al. 2004; Gay and Plant 1987) after binding to the hypothalamic NMDA receptors. We collected two additional blood samples at 15-min intervals after the NMA injection. After the experiment, animals were euthanized with an overdose of tribromoethanol. Animals were confirmed to be anestrus, and serum was stored as described above until assayed for LH.

### LHRH stimulation in pesticide-treated juvenile females

We treated a separate group of animals as described for the NMA stimulation experiment except after the third afternoon sample, we intravenously administered LHRH (25 ng). Again, two samples were collected at 15-min intervals after LHRH treatment; samples were stored as described above.

### Hormone analysis

We measured rat LH and FSH using radioimmunoassay (RIA) procedures as previously described (Hiney et al. 1996). Rat LH antiserum (NIDDK-anti-rLH-S-II), antigen (NIDDK-rLH-I-9), and reference preparation (NIDDK-rLH-RP-3) and FSH antiserum (NIDDK-rFSH-I-9) and reference preparation (NIDDK-rFSH-RP-2) were purchased from the National Institutes of Health Pituitary Hormones and Antisera Center (Harbor-UCLA Medical Center, Torrance, CA). The LH assay had a sensitivity of 0.07 ng/mL, and the FSH assay had a sensitivity of 0.4 ng/mL. We measured serum E_2_ using an RIA kit purchased from Diagnostic Products Corp. (Los Angeles, CA) as previously described (Hiney et al. 1996). The E_2_ assay sensitivity was 8.0 pg/mL. All assays had inter- and intra-assay coefficients of variation of < 10%.

### Statistical analysis

Values are expressed as the mean ± SE. We analyzed differences between treatment groups in timing of puberty using the Student’s paired *t*-test; hormone levels (morning FSH, LH, and E_2_) were analyzed by analysis of variance (ANOVA) followed by post hoc testing using Student-Newman-Keuls multiple-range test. We analyzed differences in LH serum levels among treatment groups comparing morning, afternoon, and poststimulation values by the Kruskal-Wallis test (nonparamteric ANOVA) followed by Dunn’s multiple comparisons test. We considered probability values of *p* < 0.05 to be statistically significant. We used INSTAT and PRISM software, version 3.0, for personal computer (GraphPad, San Diego, CA) to calculate and graph results.

## Results

### Effect of ESF exposure on puberty-related hormones and the onset of puberty

**Figure 1:**
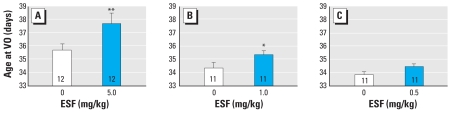
Effect of short-term oral administration of ESF at 5 mg/kg (*A*), 1 mg/kg (*B*), and 0.5 mg/kg (*C*) on the age at VO. VO was delayed by 2 days in animals treated with 5.0 mg/kg ESF (*A*) and by 1 day in animals treated with 1.0 mg/kg ESF (*B*). Values represent mean ± SE; the number within each bar indicates the number of animals. *p < 0.05, and **p < 0.01 compared with control.

Short-term exposure to ESF during juvenile development did not alter mean (± SE) daily weight gain at any dose administered. Controls gained 4.2 ± 0.11 g, whereas animals receiving 5.0, 1.0, and 0.5 mg/kg gained 4.07 ± 0.15, 4.11 ± 0.15, and 4.25 ± 0.22 g, respectively. However, the 5.0 mg/kg and 1.0 mg/kg doses of the pesticide delayed (*p* < 0.01 and *p* < 0.05, respectively) the age at VO compared with corn oil controls (Figure 1). ESF delayed the onset of puberty by 2 days in animals dosed with 5.0 mg/kg and by 1 day in females dosed with the 1.0 mg/kg compared with control animals.

We performed cytologic evaluations on each rat on their respective day of VO and determined the stage of the estrous cycle. The interval from VO to D1 was the same for pesticide-treated and control animals in which the smear on the day of VO was either proestrus or estrus (1.25 days).

**Figure 2:**
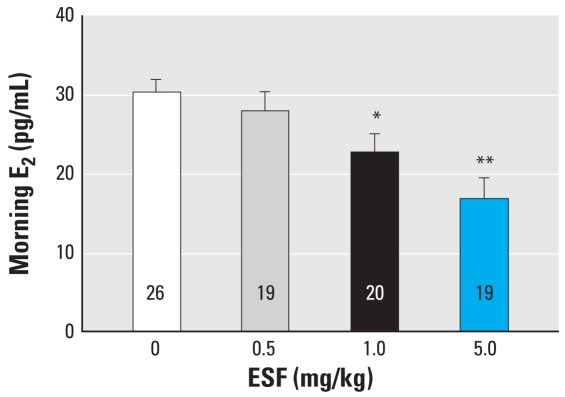
Effect of short-term oral administration of ESF on serum E_2_ in female rats on PND29. The 1.0-mg/kg dose caused a 1.3-fold decrease in serum E_2_, whereas the 5.0 mg/kg dose caused a 2-fold decrease. Values represent mean ± SE; the number within each bar indicates the number of animals. *p < 0.05, and **p < 0.01 compared with control.

**Figure 3:**
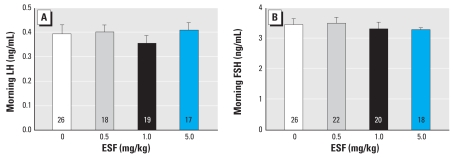
Effect of short-term oral administration of ESF on morning serum LH and FSH levels. Values represent the mean ± SE; and the number within each bar indicates the number of animals.

We then compared serum levels of the puberty-related hormones LH, FSH, and E_2_ from PND29 pesticide-treated and control animals. Animals exposed to the two highest doses of ESF (1.0 and 5.0 mg/kg) exhibited a 1.3- and 2-fold decrease in serum E_2_, respectively, compared with controls (Figure 2). Interestingly, the morning serum levels of both LH and FSH were unchanged at any dose of ESF (Figure 3).

### Effect of ESF on morning-to-afternoon basal levels of LH and hypothalamic responsiveness to NMA

**Figure 4:**
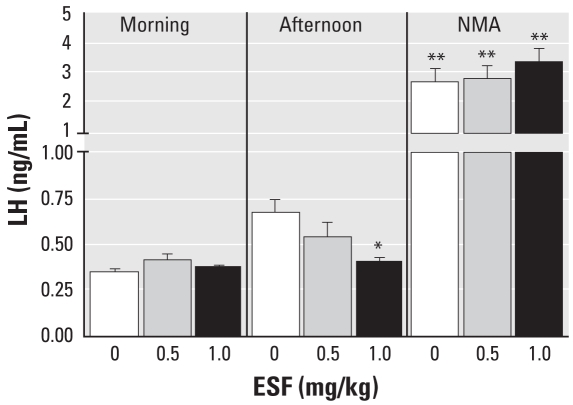
Effect of short-term oral administration of ESF on morning, afternoon, and NMA-stimulated LH release *in vivo*. Values for morning and afternoon represent the mean ± SE of three samples for each time point from each animal; post-NMA LH levels represent the peak value (number of animals: controls, *n* = 12; 0.5 mg/kg NMA, *n* = 14; 1.0 mg/kg NMA, *n* = 16). *p < 0.05 compared with control. **p < 0.05 compared with afternoon values.

Because morning LH levels were unaltered in pesticide-treated animals, we assessed whether ESF affected the morning-to-afternoon pattern of LH secretion. During peripubertal development, the release of LH becomes more prominent in the afternoon, which is a centrally mediated event (Ojeda et al. 1986). To assess whether ESF affects this pattern of LH secretion, we dosed animals from PND22 with either the pesticide (0.5 and 1.0 mg/kg) or corn oil and took morning and afternoon blood samples on PND30. Control animals exhibited a 2-fold rise in basal afternoon LH levels compared with the morning levels (*p* < 0.01). The mean afternoon LH levels in control animals were higher (*p* < 0.05) than those of animals treated with 1.0 mg/kg ESF (Figure 4).

To assess hypothalamic responsiveness, animals were injected with NMA after the third afternoon blood sample to induce LH release. NMA stimulated marked increases in LH secretion compared with their respective afternoon levels in all three groups of animals (*p* < 0.05), but we noted no differences between either ESF treatment group compared with controls. These data demonstrate that hypothalamic responsiveness to NMA stimulation was not altered by the pesticide (Figure 4).

We conducted a final experiment to assess pituitary responsiveness after ESF exposure. We dosed animals with ESF and collected serial blood samples as described above, except that instead of NMA administration, we challenged these animals with LHRH (25 ng) (Dees et al. 1985). LHRH markedly (*p* < 0.001) stimulated LH release over afternoon basal levels in control and both ESF treatment groups, demonstrating that pituitary responsiveness was not affected by the pesticide (data not shown).

## Discussion

This is the first study to show an inhibitory action of a type II pyrethroid pesticide, ESF, on the hypothalamic control of prepubertal gonadotropin secretion. This study is also the first to show that short-term administration of ESF to juvenile animals significantly delays the onset of female puberty. The dose of 1.0 mg/kg used for our short-term puberty studies was two times lower than the stated no observable effect level (NOEL) of 2.0 mg/kg/day used for the dietary developmental study in rats (U.S. EPA 1998).

We found only one *in vivo* study that evaluated puberty in female animals exposed to a type II pyrethroid. Moniz et al. (2005) dosed pregnant animals with a commercial-grade formulation (Bernardi MM, personal communication) of an unknown purity of fenvalerate. Moniz et al. (2005) found age at VO to be delayed in exposed offspring. However, commercial-grade formulations typically contain other compounds, such as solvents and petroleum distillates (Meister 1997). No conclusions can be made as to whether the effects found by Moniz et al. (2005) were due to fenvalerate or the other components in the formulation, so we cannot directly compare their results with ours.

In the present study, we dosed immature animals 1 day postweaning until just before the onset of puberty, at which time we measured morning serum levels of LH, FSH, and E_2._ Females dosed with 5.0 and 1.0 mg/kg ESF had significantly suppressed E_2_ levels.

Interestingly, in our study, ESF did not affect the morning basal secretion of FSH or LH. If ESF were acting only at the ovarian level to suppress serum E_2_ levels, a compensatory increase in LH would be expected because of the removal of the negative feedback effect at the hypothalamus. However, the morning LH levels in the pesticide-treated animals were the same as those in controls. This atypical response to the low E_2_ suggested a hypothalamic deficit.

However, our results for afternoon LH levels showed an inhibition in the afternoon hormonal rise in animals treated with even the lowest dose (0.5 mg/kg) of pesticide. The physiologic pattern of both LH and FSH secretion is periodic and intermittent, although more so for LH. Before the onset of puberty, the amplitude of LH release is low; however, once puberty begins, the release of LH becomes more prominent in the afternoon. Both rats and humans exhibit a similar release pattern (Delemarre-Van De Waal et al. 1991; Urbanski and Ojeda 1987), which is centrally driven (Knobil 1980).

Delayed puberty most commonly occurs because of decreased E_2_ resulting from either hypothalamic and/or pituitary dysfunction or from a direct effect on the ovary. Many toxicants can perturb multiple systems, and ESF may also be interfering with ovarian steroid hormone biosynthesis as well. However, if ESF acted solely on the ovary to suppress E_2_ synthesis or release, the expected response would be a compensatory increase in LH levels because of the feedback mechanism from the ovary to the hypothalamus and pituitary. Morning LH levels in the pesticide-treated animals were the same as those in control animals, indicating an abnormal feedback response.

When we measured LH in serial morning and afternoon blood samples, the afternoon rise in LH was suppressed in females dosed at even the lowest ESF concentration. Based on the findings of low serum E_2_ and low afternoon LH, we expected the ESF-treated animals to release less LH than controls after NMA stimulation. However, the ESF-treated animals responded to NMA stimulation as well as the controls.

The toxic effects of pyrethroids are caused by the prolongation of the open state of voltage-dependent sodium channels, resulting in repetitive firing of the neurons. If the delay in puberty was due to the action of the pesticide on the sodium channels of the LHRH neurons, the expected result would be an initial increase in LH due to the rapid release of LHRH, followed by a decrease in LH levels after the releasable pool of LHRH was exhausted.

One possible site of pyrethroid action that could potentially cause the delay in puberty is the NMDA receptor; activation of the NMDA receptor has been suggested as a mediator of pyrethroid neurotoxicity (Chugh et al. 1992). Furthermore, Wu and Liu (2003) found that both c-fos and c-jun were expressed in adult male rats exposed to deltamethrin. Blocking the NMDA receptor with the antagonist MK-801 eliminated c-fos and c-jun expression, suggesting that the pesticide can act through this excitatory amino acid (EAA) pathway. A possible explanation of our data is that ESF may be interfering with hypothalamic storage, release, and/or transport of the EAAs.

Any or all of the EAAs, such as glutamate or aspartate, may not be present in high enough levels to fully activate the NMDA receptor. Thus, when we administered the NMDA receptor agonist, more unoccupied receptor sites were available for binding and subsequent stimulation of LHRH release. Other neurotransmitters, such as norepinephrine, may also be adversely affected by the pesticide.

Although the exact mechanism of action is unknown at this time, we observed the effects at dosage levels below the NOEL established through chronic dietary exposure studies in rats. The U.S. EPA (1998) stated that “There is no evidence of additional sensitivity to young rats or rabbits following pre- or post-natal exposure to esfenvalerate.” The present study shows that immature female rats exposed to 1.0 mg/kg/day are sensitive to this pesticide, as evidenced by their delay in the onset of puberty. Delayed pubertal onset in humans has been associated with low bone mass density (Ho and Kung 2005), and estrogen is necessary for bone mineral acquisition in both girls and boys (Yilmaz et al. 2005). Importantly, a lowered endogenous estrogen level in females is one factor associated with bone fragility (Hoffman and Bradshaw 2003).

This could potentially affect current established exposure levels for humans, because the reference dose for ESF of 0.02 mg/kg/day is based directly on the rodent NOEL of 2.0 mg/kg/day. Obviously, more basic research is needed in this area; because of the worldwide use of this class of pesticide, further studies are warranted.

## References

[b1-ehp-116-1243] Andersen H, Vinggaard A, Rasmussen T, Gjermandsen I, Bonefeld-Jorgensen E (2002). Effects of currently used pesticides in assays for estrogenicity, androgenicity, and aromatase activity in vitro. Toxicol Appl Pharmacol.

[b2-ehp-116-1243] Bourguignon JP, Gerard A, Mathieu J, Mathieu A, Franchimont P (1990). Maturation of the hypothalamic control of pulsatile gonadotropin-releasing hormone secretion at the onset of puberty. I. Increased activation of *N*-methyl-d-aspartate receptors. Endocrinology.

[b3-ehp-116-1243] Bouwman H, Sereda B, Meinhardt H (2006). Simultaneous presence of DDT and pyrethroid residues in human breast milk from a malaria endemic area in South Africa. Environ Pollut.

[b4-ehp-116-1243] Casida J, Quistad G (1998). Golden age of insecticide research: past, present, or future?. Annu Rev Entomol.

[b5-ehp-116-1243] CDC (2005). Third National Report on Human Exposure to Environmental Chemicals.

[b6-ehp-116-1243] Chen H, Hu G, Xiao J, Zhou J, Xiao H, Wang X (2002). Estrogenicity of organophosphorus and pyrethroid pesticides. J Toxicol Environ Health Part A.

[b7-ehp-116-1243] Chugh Y, Sankaranarayanan A, Sharma P (1992). MK-801 antagonizes the lethal action of centrally and peripherally administered cypermethrin in mice and rats. J Pharm Pharmacol.

[b8-ehp-116-1243] Claypool LE, Kasuya E, Saitoh Y, Marzban F, Terasawa E (2000). *N*-Methyl d,l-aspartate induces the release of luteinizing hormone releasing hormone in the prepubertal and pubertal female rhesus monkey as measured by *in vivo* push-pull perfusion in the stalk median eminence. Endocrinology.

[b9-ehp-116-1243] Colborn T, vom Saal F, Soto A (1993). Developmental effects of endocrine-disrupting chemicals in wildlife and humans. Environ Health Perspect.

[b10-ehp-116-1243] Dees WL, Rettori V, Kozlowski G, McCann S (1985). Ethanol and the pulsatile release of luteinizing hormone, follicle stimulating hormone and prolactin in ovariectomized rats. Alcohol.

[b11-ehp-116-1243] Dees WL, Skelley CW (1990). Effects of ethanol during the onset of female puberty. Neuroendocrinology.

[b12-ehp-116-1243] Delemarre-Van De Waal H, Wennink J, Odink R (1991). Gonadotropin and growth hormone secretion throughout puberty. Acta Paediatr Scand.

[b13-ehp-116-1243] Dissen G, Dearth R, Scott H, Ojeda S, Dees W (2004). Alcohol alters luteinizing hormone secretion in immature female rhesus monkeys by a hypothalamic action. Endocrinology.

[b14-ehp-116-1243] EXTOXNET (1996). Extension Toxicology Network. Pesticide Information Profiles: Esfenvalerate.

[b15-ehp-116-1243] FDA Pesticide Program Residue Monitoring 1993–2003.

[b16-ehp-116-1243] Freeman N, Shalat S, Black K, Jimenez M, Donnelly K, Calvin A (2004). Seasonal pesticide use in a rural community on the US/Mexico border. J Expo Anal Environ Epidemiol.

[b17-ehp-116-1243] Gay V, Plant T (1987). *N*-Methyl-d,l-aspartate elicits hypothalamic gonadotropin-releasing hormone release in prepubertal male rhesus monkeys (*Macaca mulatta*). Endocrinology.

[b18-ehp-116-1243] Go V, Garey J, Wolff M, Pogo B (1999). Estrogenic potential of certain pyrethroid compounds in the MCF-7 human breast carcinoma cell line. Environ Health Perspect.

[b19-ehp-116-1243] Gore A, Wu T, Rosenberg J, Roberts J (1996). Gonadotropin-releasing hormone and NMDS receptor gene expression and colocalization change during puberty in female rats. J Neurosci.

[b20-ehp-116-1243] Harms PG, Ojeda SR (1974). Method for cannulation of the rat jugular vein. J Appl Physiol.

[b21-ehp-116-1243] Heudorf U, Angerer J (2001). Metabolites of pyrethroid insecticides in urine specimens: current exposure in an urban population in Germany. Environ Health Perspect.

[b22-ehp-116-1243] Hiney JK, Srivastava V, Nyberg CL, Ojeda SR, Dees WL (1996). Insulin-like growth factor-1 of peripheral origin acts centrally to accelerate the initiation of female puberty. Endocrinology.

[b23-ehp-116-1243] Ho A, Kung A (2005). Determinants of peak bone mineral density and bone area in young women. J Bone Miner Metab.

[b24-ehp-116-1243] Hoffman B, Bradshaw K (2003). Delayed puberty and amenorrhea. Semin Reprod Med.

[b25-ehp-116-1243] Institute of Laboratory Animal Resources (1996). Guide for the Care and Use of Laboratory Animals.

[b26-ehp-116-1243] Kasat K, Go V, Pogo B (2002). Effects of pyrethroid insecticides and estrogen on WNT10B protooncogene expression. Environ Int.

[b27-ehp-116-1243] Kim Y, Shin J, Kim H, Lee S, Kang I, Kim T (2004). Assessing estrogenic activity of pyrethroid insecticides using in vitro combination assays. J Reprod Dev.

[b28-ehp-116-1243] Knobil E (1980). The neuroendocrine control of the menstrual cycle. Recent Prog Horm Res.

[b29-ehp-116-1243] Kunimatsu T, Yamada T, Ose K, Sunami O, Kamita Y, Okuno Y (2002). Lack of (anti-) androgenic or estrogenic effects of three pyrethroids (esfenvalerate, fenvalerate, and permethrin) in the Hershberger and uterotrophic assays. Regul Toxicol Pharmacol.

[b30-ehp-116-1243] Lemaire G, Mnif W, Mauvvais P, Balaguer P, Rahmani R (2006). Activation of α- and β-estrogen receptors by persistent pesticides in reporter cell lines. Life Sci.

[b31-ehp-116-1243] Lu C, Baarr D, Pearson M, Bartell S, Bravo R (2006). A longitudinal approach to assessing urban and suburban children’s exposure to pyrethroid pesticides. Environ Health Perspect.

[b32-ehp-116-1243] Meister RT (1997). Farm Chemicals Handbook.

[b33-ehp-116-1243] Moniz A, Cruz-Casallas P, Salzgeber S, Varoli F, Spinosa H, Bernardi M (2005). Behavioral and endocrine changes induced by prenatal fenvalerate exposure in female rats. Neurotoxicol Teratol.

[b34-ehp-116-1243] Nyberg C, Hiney J, Minks J, Dees WL (1993). Ethanol alters *N*-methyl-dl-aspartic acid-induced secretion of luteinizing hormone releasing hormone and the onset of puberty in the female rat. Neuroendocrinology.

[b35-ehp-116-1243] OECD (1997). Draft Detailed Review Paper: Appraisal of Test Methods for Sex-Hormone Disrupting Chemicals.

[b36-ehp-116-1243] Ojeda S, Urbanski H, Ahmed C (1986). Onset of female puberty: studies in the rat. Recent Prog Horm Res.

[b37-ehp-116-1243] Roberts T, Hutson D (1999). Metabolic Pathways of Agrochemicals, Part 2. Insecticides and Fungicides.

[b38-ehp-116-1243] Saito K, Tomigahar Y, Ohe N, Isobe N, Nakatsuka I, Kaneko H (2000). Lack of significant estrogenic or antiestrogenic activity of pyrethroid insecticides in three in vitro assays based on classic estrogen receptor alpha-mediated mechanisms. Toxicol Sci.

[b39-ehp-116-1243] Smyth C, Wilkinson M (1994). A critical period for glutamate receptor-mediated induction of precocious puberty in female rats. J Neuroendocrinol.

[b40-ehp-116-1243] Soderlund DM, Clark JM, Sheets LP, Mullin LS, Piccirillo VJ, Sargent D (2002). Mechanisms of pyrethroid neurotoxicity: implications for cumulative risk assessment. Toxicology.

[b41-ehp-116-1243] Tulve N, Jones P, Nishioka M, Fortmann R, Croghan C, Zhou J (2006). Pesticide measurements from the first national environmental health survey of child care centers using a multi-residue GC/MS analysis method. Environ Sci Technol.

[b42-ehp-116-1243] Urbanski HF, Ojeda SR (1987). Activation of luteinizing hormone-releasing hormone release advances the onset of female puberty. Neuroendocrinology.

[b43-ehp-116-1243] U.S. EPA (1997). Special Report on Environmental Endocrine Disruption: An Effect Assessment and Analysis.

[b44-ehp-116-1243] U.S. EPA (U.S. Environmental Protection Agency) (1998). Esfenvalerate; pesticide tolerances. Final rule. Fed Reg.

[b45-ehp-116-1243] U.S. EPA (U.S. Environmental Protection Agency) (2001). Pesticide Industry Sales and Usage: 2000 and 2001 Market Estimates.

[b46-ehp-116-1243] Vijverberg HP, van den Bercken J (1990). Neurotoxicological effects and the mode of action of pyrethroid insecticides. Crit Rev Toxicol.

[b47-ehp-116-1243] Wu A, Liu Y (2003). Prolonged expression of c-Fos and c-Jun in the cerebral cortex of rats after deltamethrin treatment. Brain Res Mol Brain Res.

[b48-ehp-116-1243] Yilmaz D, Ersoy B, Bilgin E, Gumuser G, Onur E, Pinar E (2005). Bone mineral density in girls and boys at different pubertal stages: relation with gonadal steroids, bone formation markers, and growth parameters. J Bone Miner Metab.

